# Supplementary material for: Global quantification of the dispersion effect with POLDER satellite data
Source: Nat Commun. 2025 Aug 2;16:7087. doi: 10.1038/s41467-025-62238-3 (PMC12317032; doi:10.1038/s41467-025-62238-3)
Supplement: Supplementary file 1 — Supplementary Information [file 41467_2025_62238_MOESM1_ESM.pdf]

Supplementary information for

Global Quantification of the Dispersion Effect with POLDER Satellite Data

Hengqi Wang[1,2], Yiran Peng[1*], Antonio Di Noia[3], Huazhe Shang[2], Husi Letu[2], Bastiaan van Diedenhoven[4], Otto P. Hasekamp[4], Yangang Liu[5], and Johannes Quaas[6]

[1]Department of Earth System Science, Ministry of Education Key Laboratory for Earth System Modeling, Institute for Global Change Studies, Tsinghua University, Beijing, China

[2]State Key Laboratory of Remote Sensing and Digital Earth, Aerospace Information Research Institute, Chinese Academy of Sciences, Beijing, China

[3]Institute of Environmental Physics, University of Bremen, Bremen, Germany

[4]SRON Space Research Organisation Netherlands, Leiden, Netherlands

[5]Environmental Science and Technologies Department, Brookhaven National Laboratory, Upton, New York, USA

[6]Leipzig Institute of Meteorology, Leipzig University, Leipzig, Germany

Corresponding author: Yiran Peng (pyiran@mail.tsinghua.edu.cn)

CONTENTS

Supplementary Figures **3**

Supplementary Fig. 1: The histogram depicts the frequency distribution for parameter b 3

Supplementary Fig. 2: The spatial distribution of the relevant variables calculated to explain the spatial distribution of parameter b. 4

Supplementary Fig. 3: The counts and corresponding percentages of cloud types 5

Supplementary Tables **6**

Supplementary Table 1: Impacts of different sources of uncertainty on parameter b 6

Supplementary Table 2: Impacts of different sources of uncertainty on parameter b for land and ocean scales. 7

Supplementary Figures

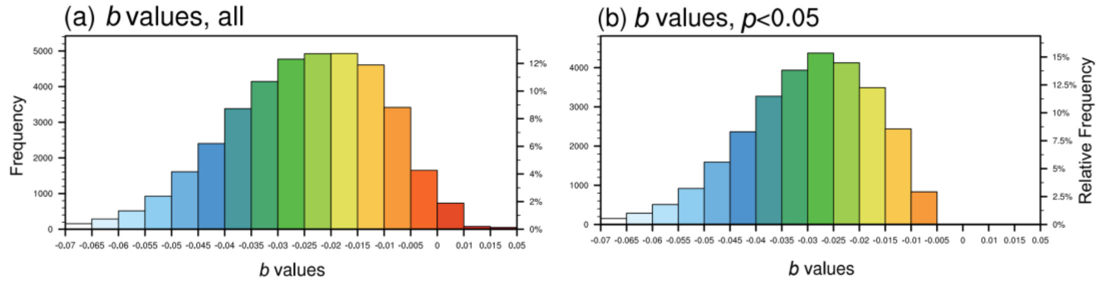

Supplementary Fig. 1: The histogram depicts the frequency distribution for parameter *b*. (a) All data and (b) selected data with p < 0.05. The left vertical axis represents frequency, the right vertical axis represents relative frequency, and warmer colors indicate a more positive *b* value.

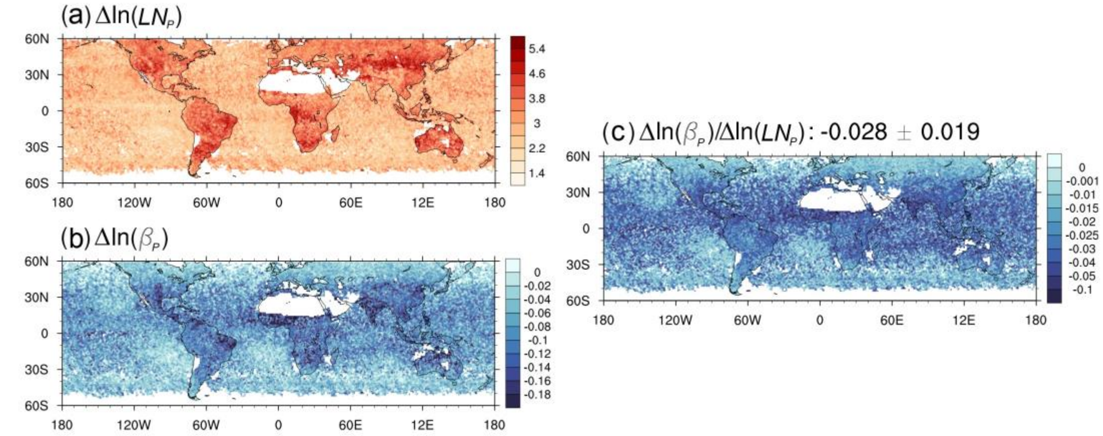

Supplementary Fig. 2: The spatial distribution of the relevant variables calculated to explain the spatial distribution of parameter $\boldsymbol{b}$**.** (a) The difference between the mean of the upper 10% and the lower 10% of the liquid water content per cloud droplet (LN_P) within the grid (i.e., $\Delta\ln(LN_P)$), (b) the corresponding difference in the mean of the particle size distribution parameter (β_P) within the grid (i.e., $\Delta\ln(\beta_P)$), and (c) the ratio of the two (i.e., $\Delta\ln(\beta_P)/\Delta\ln(LN_P)$).

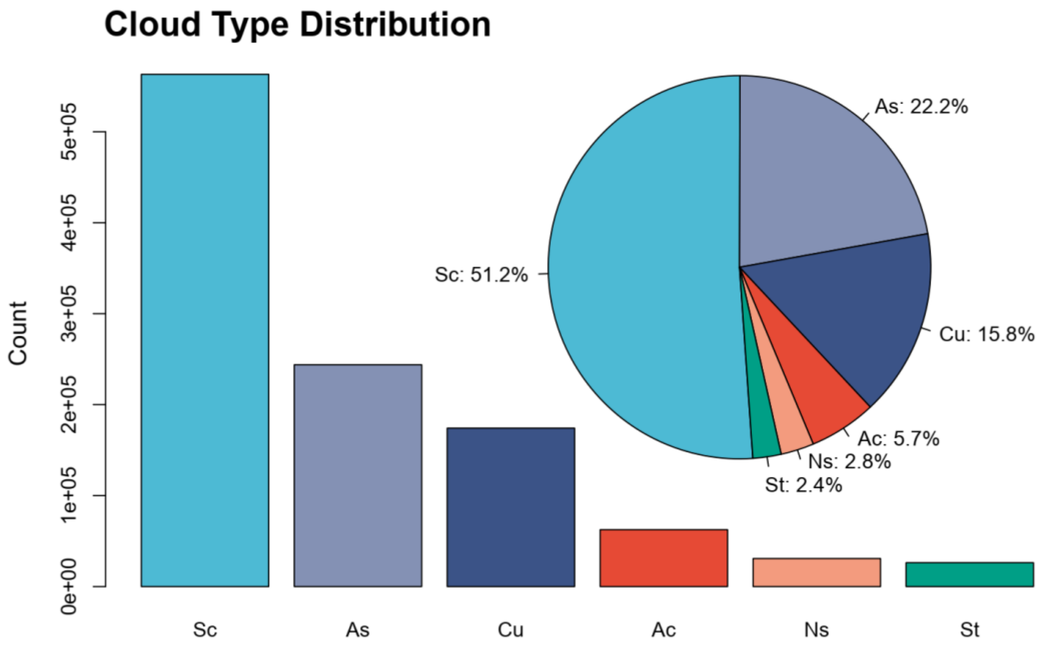

Supplementary Fig. 3: The counts and corresponding percentages of cloud types. The data are categorized following the International Satellite Cloud Climatology Project (ISCCP) classification scheme, based on the cloud top pressure (p_t) and cloud optical depth (τ_c). Specifically, Cu refers to cumulus ($680 \text{ hpa} < p_t \leq 1025 \text{ hpa}$, $0 < \tau_c \leq 3.55$), Sc to stratocumulus ($680 \text{ hpa} < p_t \leq 1025 \text{ hpa}$, $3.55 < \tau_c \leq 22.63$), St to stratus ($680 \text{ hpa} < p_t \leq 1025 \text{ hpa}$, $22.63 < \tau_c \leq 450$), Ac to altocumulus ($440 \text{ hpa} < p_t \leq 680 \text{ hpa}$, $0 < \tau_c \leq 3.55$), As to altostratus ($440 \text{ hpa} < p_t \leq 680 \text{ hpa}$, $3.55 < \tau_c \leq 22.63$), and Ns to nimbostratus ($440 \text{ hpa} < p_t \leq 680 \text{ hpa}$, $22.63 < \tau_c \leq 450$).

Supplementary Tables

Supplementary Table 1: Impacts of different sources of uncertainty on parameter b. $Bias$ and $RMSE$ represent the deviation and root mean square error introduced by different sources in the retrieval of the effective radius (R_e) and effective variance (V_e). Variations in sources and fitting methods introduce uncertainty into the calculation of parameter b, denoted as b_{S_F} and SE_{S_F}, where S represents different sources, and F indicates the fitting method, with d for the direct method and p for the pre-binned method. These values correspond to the estimated b considering each source of uncertainty and its associated standard error (SE). The final row presents the ensemble uncertainty considering all sources, providing the best estimate and the 5 ~ 95% confidence interval.

Source and method	R_e	V_e	Fitting method	
			Direct	Pre-binned
Src1: Limitations of the POLDER-NNs	$Bias = 0.08$<br>$RMSE = 0.92$	$Bias = -0.01$<br>$RMSE = 0.03$	$b_{s1\_d} = -0.026$<br>$SE_{s1\_d} = 0.00004$	$b_{s1\_p} = -0.021$<br>$SE_{s1\_p} = 0.0014$
Src2: Heterogeneity of clouds	$Bias = -0.71$<br>$RMSE = 0.88$	$Bias = 0.02$<br>$RMSE = 0.04$	$b_{s2\_d} = -0.029$<br>$SE_{s2\_d} = 0.00005$	$b_{s2\_p} = -0.024$<br>$SE_{s2\_p} = 0.0014$
Src3: Retrieval method, wavelength, grid scale	/	/	$b_{s3\_d} = -0.022$<br>$SE_{s3\_d} = 0.0002$	$b_{s3\_p} = -0.021$<br>$SE_{s3\_p} = 0.007$
Ensemble via a Monte Carlo method	/	/	$b = -0.024\ [-0.026 \sim -0.022]$	

Supplementary Table 2: Impacts of different sources of uncertainty on parameter b for land and ocean scales. Src1 to Src3 represent different sources. Variations in sources and fitting methods introduce uncertainty into the calculation of parameter b, denoted as b_{S_F} and SE_{S_F}, where S represents different sources (Src1 ~ Src3), and F indicates the fitting method, with d for the direct method and p for the pre-binned method. These values correspond to the estimated b considering each source of uncertainty and its associated standard error (SE). The final row presents the ensemble uncertainty considering all sources, providing the best estimate and the 5 ~ 95% confidence interval.

Source	Land		Ocean	
	Direct	Pre-binned	Direct	Pre-binned
Src1	$b_{s1\_d} = -0.026$<br>$SE_{s1\_d} = 0.00006$	$b_{s1\_p} = -0.023$<br>$SE_{s1\_p} = 0.0024$	$b_{s1\_d} = -0.030$<br>$SE_{s1\_d} = 0.00007$	$b_{s1\_p} = -0.025$<br>$SE_{s1\_p} = 0.0013$
Src2	$b_{s2\_d} = -0.029$<br>$SE_{s2\_d} = 0.00007$	$b_{s2\_p} = -0.025$<br>$SE_{s2\_p} = 0.0027$	$b_{s2\_d} = -0.034$<br>$SE_{s2\_d} = 0.00008$	$b_{s2\_p} = -0.028$<br>$SE_{s2\_p} = 0.0014$
Src3	$b_{s3\_d} = -0.023$<br>$SE_{s3\_d} = 0.0006$	$b_{s3\_p} = -0.023$<br>$SE_{s3\_p} = 0.010$	$b_{s3\_d} = -0.030$<br>$SE_{s3\_d} = 0.0003$	$b_{s3\_p} = -0.025$<br>$SE_{s3\_p} = 0.008$
Ensemble	$b = -0.025\ [-0.028 \sim -0.022]$		$b = -0.029\ [-0.031 \sim -0.026]$	
